# Validating a Short Version of the External Food Cue Responsiveness Scale (EFCR-6) in Preadolescents: Initial Evidence on Validity and Reliability

**DOI:** 10.21203/rs.3.rs-5976498/v1

**Published:** 2025-12-16

**Authors:** Zhuoya Zhang, Dabin Yeum, Timothy J. Renier, Delaina Carlson, Diane Gilbert-Diamond, Jennifer A. Emond

**Affiliations:** Dartmouth College; University of Washington; Dartmouth College; University of Connecticut; Dartmouth College; Dartmouth College

**Keywords:** external food cue responsiveness, external eating, food responsiveness, preadolescent, childhood obesity

## Abstract

**Background:**

Responsivity to external food cues is linked to higher adiposity in children, yet validated tools for middle childhood are lacking. This study examined the factor structure of the External Food Cue Responsiveness (EFCR) scale in 185 US preadolescents.

**Methods:**

Parents completed the original nine-item EFCR scale. Exploratory factor analysis (EFA) was conducted to evaluate the factor structure. Internal consistency was assessed via Cronbach’s *α* and McDonald’s. One-year test-retest reliability was evaluated via intraclass correlation (ICC, N = 96). Convergent validity was examined via Spearman’s correlation of the reduced scale with food approach appetitive traits (Child Eating Behavior Questionnaire), lab-observed eating in the absence of hunger (EAH), and BMI-z. Construct validity was assessed via Spearman’s correlations with child media use, trait impulsiveness, and parenting style.

**Results:**

EFA revealed a two-factor structure with six items (EFCR-6) (RMSEA = 0.06, SRMR = 0.05, CFI = 0.98, TLI = 0.96, loading range: 0.43–0.66), representing visual and auditory cue responsiveness. A global EFCR-6 score converged with multiple food approach appetitive traits (*r* range 0.24–0.41, *p* < .01) and was associated with child media use, coercive feeding, and parenting style (*p* < .05). The EFCR-6 scale demonstrated good internal consistency (*α* = 0.74) and one-year test-retest reliability (ICC = 0.72).

**Conclusions:**

The six-item EFCR scale shows promise as a valid and reliable measure of external food cue responsivity in preadolescents. Cross-validation in independent samples is recommended.

## INTRODUCTION

Childhood obesity is a significant public health concern. The prevalence has been consistently rising since 1999, and currently, about 20% of US children aged 6 to 11 years have obesity (1). Children with overweight and obesity are at higher risk of physical and mental health conditions (2). Further, childhood obesity can persist to adulthood and accelerate the development of health conditions later in life (3). Identifying modifiable risk factors of childhood obesity and taking preventative actions would benefit children’s long-term health.

Heightened responsivity to external food cues may promote hedonic eating and weight gain in children. Food cues are prevalent in the current obesogenic food environment. Cues can be sensory (e.g., seeing a restaurant logo, smelling foods), social (e.g., hearing people talking about foods on social media), and environmental (e.g., being in a restaurant or time of the day) (4). Constant exposure to these external cues may promote cued food seeking and consumption (5). As outlined in the Cue-Influencer-Reactivity-Outcome (CIRO) model, repeated exposures to external food cues, such as food marketing, paired with the rewarding value of consuming foods, may train a learned responsiveness of hedonic eating after cue exposure (4). Food approach appetitive traits relate to one’s general desire to eat (6). Greater food approach, such as food responsiveness (FR), has been consistently linked to higher adiposity in children under 13 years (7) and undesirable cardiometabolic health profiles in adolescents (8). Understanding the etiology of external food cue responsiveness (EFCR) in children will inform efficacious childhood obesity prevention and intervention efforts.

An interplay of child and family factors shapes children’s propensity to react to food cues. Several studies found that the genetic risk of obesity is associated with food responsiveness and eating in the absence of hunger (EAH) in children (9–11). Renier and colleagues observed a 1% to 7% increase in FR per standard deviation increase in four polygenic risk scores (PRSs) in children aged 9 to 12 years (9). Second, child disposition and lifestyle behaviors can affect food cue reactivity. Studies found that child inhibitory control (standardized = −0.30, *p* = 0.008) (12) and frequent TV watching (= 0.38, *p* < 0.001) (13) were linked to child reactivity to external food cues. Lastly, household environment and parenting behaviors can shape a child’s appetitive traits. More chaos at home was related to higher food responsiveness (*r* = 0.21, *p* < 0.001) in preschoolers (14). Authoritarian parenting (= 0.17, *p* = 0.007) (15) and instrumental feeding (i.e., using food as rewards) (= 0.11, *p* < 0.05) (16) positively correlated with FR in children. Therefore, the development of food cue responsivity is complex and correlates with individual and family factors.

Middle childhood (age 6 to 12 years) is critical to developing reactivity to external food cues. First, as working memory expands in this developmental stage, children become more receptive to environmental stimuli, such as logos and advertisements (17,18). Children in this age group also have more autonomy in food choices and more exposure to food cues outside the household, such as schools and social gatherings (19). They may thus develop conditioned eating responses to these external cues, which may override internal hunger cues and lead to hedonic eating. Importantly, research shows that food responsiveness established in childhood may persist into adolescence and affect their obesity risk (20). Assessing a child’s reaction to external food-related stimuli is crucial to guiding clinical practices and interventions, yet validated instruments are lacking for this age population.

The External Food Cue Responsiveness (EFCR) Scale is a parent-report questionnaire initially developed for assessing responsivity to environmental food cues in preschoolers (13). The scale contains nine items encompassing child behavioral responses to sensory (e.g., “My child gets excited by the sound of food cooking”), social (e.g., “My child asks for foods or drinks that other kids eat”), and environmental cues (e.g., “My child points out snack or drink vending machines”) (Supplementary Table S1). The scale resulted from a confirmatory factor analysis (CFA) with items reflecting young children’s reaction to external food cues identified by a focus group of parents of preschool-age children. Another commonly used instrument on child external eating is the Children’s Dutch Eating Behavior Questionnaire (DEBQ-c) (21). The DEBQ-c has a subscale on external eating (EE, five items, = 0.74). While the DEBQ-EE examines some visual and olfactory cues (e.g., “Desire to eat when see or smell food”), its questions overlook cues distinct to the contemporary food environment, such as branded packaging and food placement. The EFCR scale extends existing metrics by assessing children’s reactions to child-directed food marketing (e.g., “My child likes certain snacks because of the packaging”). Additionally, the scale evaluates children’s response to social food cues (e.g., “My child wants to eat when people talk about food”), as past research highlights the role of peers in influencing children’s food choices (22). The EFCR questionnaire showed high internal validity (Cronbach’s = 0.86) and converged with CEBQ-FR (*r* = 0.68; *p* < 0.001) in children aged 2 to 5 years (13). However, its validity for children in middle childhood has not been examined. Further, the scale has not been tested against observed measures of externally cued eating, such as EAH. Test-retest reliability was also not established.

This current study aimed to assess the factor structure and one-year test-retest reliability of the EFCR scale in children aged 9 to 12 years. Additionally, we examined its convergent validity against CEBQ, EAH, and weight status and face validity against correlates of food responsivity. We hypothesized that the EFCR scale would positively correlate with the CEBQ food approach appetitive traits (i.e., food responsiveness, emotional overeating, and enjoyment of food), EAH, and BMI-z. Further, we hypothesized that EFCR would positively correlate with obesity polygenic risk scores (PRSs), child trait impulsivity, child media use, household chaos, and parental restrictive and instrumental feeding. Lastly, we hypothesized that EFCR would negatively correlate with demanding and responsive parenting styles.

## METHODS

### Study Sample

Data came from a longitudinal study in preadolescents (National Institutes of Health grant 1R01HD092604) (12). The study examined the association between dynamic food advertisement exposure, neural reward reactivity, and lab-observed snacking behavior in children. Children aged 9–12 years were recruited via paper flyers, email listservs, events, and word of mouth in New Hampshire and Vermont, USA, from 2019 to 2023. Children were ineligible if they had food allergies or restrictions, psychiatric or neurological disorders, appetite- or attention-altering disorders, could not comprehend English, or if their immediate family members had participated in this research. As the parent study included magnetic resonance imaging (MRI), children were excluded if they had any MRI contraindications. Children provided written assent. Parents provided written informed consent. Dartmouth College’s Committee for the Protection of Human Subjects approved all study procedures (CPHS30723).

This current work is a secondary analysis of baseline data (N = 185), including (1) parent responses to online surveys, (2) a lab-observed measure of hedonic eating, eating in the absence of hunger, (3) lab-measured child anthropometry, and (4) a saliva sample for genotyping. Test-retest reliability was assessed with parental responses on the EFCR scale collected at the one-year follow-up visit (N = 96).

### External Food Cue Responsiveness Scale, EFCR

Parents completed the External Food Cue Responsiveness scale at baseline (N = 185) and one-year follow-up (N = 96). The EFCR scale was initially developed for preschool-aged children and, in part, framed on young children’s reactions to heavily marketed, child-directed foods and drinks (13). The original scale has nine items, anchored on four-point Likert scales from “1: Never” to “4: Often.” For example, “My child wants snacks at check-out aisles” and “My child likes certain snacks because of the packaging” (Supplementary Table S1). The final score is the average of responses across items (range: 1 to 4). Higher scores reflect stronger responsivity to external food cues. The scale demonstrated high internal consistency (= .78, McDonald’s = .78) and acceptable one-year test-retest reliability in our sample (intraclass correlation, ICC = 0.76, 95% CI: 0.64, 0.84) (Supplementary Table S1).

### Convergent Validity Variables

The convergent validity analysis considered food approach appetitive traits assessed via the Child Eating Behavior Questionnaire (CEBQ), lab-observed EAH, and BMI, as prior research showed that these variables were positively linked to external food cue responsiveness (13).

Child food approach appetitive traits were assessed via the Child Eating Behaviors Questionnaire (6). Parents read Likert-scale items describing attributes of a child’s eating and rated how applicable each is to their children from 1 (never) to 5 (always). This study considered three subscales: food responsiveness (FR, four items, e.g., “If allowed to, my child would eat too much.”), emotional overeating (EOE, four items, e.g., “My child eats more when worried.”), and enjoyment of food (EF, four items, e.g., “My child loves food.”). Our analysis excluded the desire to drink subscale because the items are ambiguous on the type of beverages consumed, i.e., water vs. sweetened (9). The CEBQ subscales showed high internal consistency in our sample, with Cronbach’s values > 0.8 (Supplementary Table S2). Subscale scores are the average responses across items. Possible scores range from 1 to 5. Higher scores reflect the more parent-perceived presence of the appetitive trait.

Children participated in two eating in the absence of hunger experiments during lunch (11 a.m. to 1 p.m.) or dinner time (5 to 7 p.m.). Children were first served a pre-load meal designed to provide 30% of their daily estimated energy requirement. Next, they were provided with open bowls of snacks (gummy bears, 240 g ± 1 g, goldfish crackers, 76 g ± 0.5 g, and grapes, 235 g ± 1 g, and water) to eat ad-libitum while watching a TV show embedded with food (experimental condition) and toy commercials (control condition). Research staff weighed the snacks before and after the EAH experiment to compute the child’s caloric intake. EAH was operationalized as the difference in caloric intake between the food advertisement and toy advertisement conditions to assess food cue-induced EAH.

Trained research assistants measured the weight and height of the children three times during the baseline in-person visit. We calculated the age- and sex-adjusted body mass index (BMI) z-scores and percentiles with the CDC 2000 growth charts (24).

### Correlates of External Food Cue Responsiveness

This analysis considered child trait impulsiveness, parental feeding style, general parenting, household chaos, child media usage, and child obesity genetic risk as correlates of EFCR, as earlier research found that they were associated with external eating or food responsiveness (12,13,16).

Child trait impulsivity was assessed via the impulsiveness subscale of Eysenck’s Impulsiveness Questionnaire, a validated child-report instrument on behavioral control and capacity for delaying gratification (25). Children rated themselves on 23 yes-no questions, describing attributes of dispositional impulsivity. For example, “Do you often buy things on impulse?” or “Do you sometimes break the rules on the spur of the moment?” Final scores averaged item responses (range: 0 to 1), with higher scores suggesting greater trait impulsiveness. This questionnaire showed a high internal consistency of = .81 and .81 in our sample (Supplementary Table S2).

Feeding style was assessed via the Comprehensive Feeding Practices Questionnaires (CFPQ), adapted for families with 10- to 12-year-old children (26). Our study considered two subscales from the questionnaire: (1) restriction for weight control, i.e., parents control the child’s food intake for weight loss (eight items, = .82, .83), and (2) using food as a reward for behavior, i.e., parents offer the child their favorite foods to promote certain behaviors (two items, = 0.81, .81). Parents rated each item on a five-point Likert scale from 1 (disagree) to 5 (agree). We computed subscale scores by averaging item responses (range: 1 to 5). Higher scores suggest the more parent-perceived presence of that feeding practice.

Children assessed their parents’ parenting style with the Authoritative Parenting Index (API) (27). Our study included five items from the responsiveness subscale (e.g., “He/She likes me just the way I am,” = .75, .75) and four items from the demandingness subscale (e.g., “He/She checks to see if I do my homework,” = .52, .53) to reduce the participant burden. Children rated how well each item describes their parents on a Likert scale from 1 (not like them) to 4 (just like them). Subscale scores are the sum of ratings across items (range: 4 to 16 for demandingness; 5 to 20 for responsiveness). Based on prior research (28), we dichotomized the subscale scores at the medians and categorized parents into four parenting styles: authoritative (high demandingness and responsiveness), authoritarian (high demandingness and low responsiveness), permissive (low demandingness and high responsiveness), and neglectful (low demandingness and low responsiveness).

Household chaos was assessed via the Confusion, Hubbub, and Order Scale (CHAOS) (29). The scale has 15 items measuring attributes of orderliness and structure at home. Example items are “The atmosphere in our home is calm (reverse scored),” “We almost always seem to be rushed,” and “There is often a fuss going on at our home).” Parents rated how well each item describes their household on a Likert scale from 1 (not at all) to 4 (very much). The final score is the sum across item responses that ranges from 15 to 60. Higher scores indicate a more chaotic home environment. The scale showed good internal validity in our sample (= .87, .87) and test-retest reliability (*r* = 0.74) in the original study (29).

Parents reported child media usage on typical weekdays and weekends separately in the baseline questionnaire. They were instructed to consider time spent on all screen media devices for non-school related use. The average daily screen time was computed by weighing the weekday and weekend media use by 5/7 and 2/7, respectively.

DNA from children’s saliva samples was genotyped with the Illumina Global Screening Array 24 v1.0 or v3.0 (30). The procedures for genotyping single nucleotide polymorphism (SNP) and computing the polygenic risk scores (PRSs) have been described previously (9). This analysis considered three weighted polygenic risk scores: (1) the 97 SNP PRS (31), (2) 265 out of 295 SNPs pediatric-specific PRS (32), and (3) the ~2 million SNP PRS (33), which were shown to positively correlate with child obesogenic appetitive traits and BMI in prior research (9). The PRSs were standardized in all statistical models.

### Covariates

Parents reported on sociodemographic characteristics, including child age, sex, race/ethnicity, household annual income (i.e., less than $25,000, $25,000-$65,000, $65,000-$145,000, $145,000-$225,000, over $225,000, or prefer not to answer), and parent education (i.e., grade 8 or less, some high school, high school graduate, some post-high school, associate’s degree, bachelor’s degree, professional school or graduate school, or prefer not to answer). European ancestry was determined via genotyping.

### Statistical Analysis

Descriptive statistics were computed for the EFCR scale and covariates. We calculated counts and proportions for categorical variables and means and SDs for continuous variables. Prior research proposed that the sample size needs at least ten times the number of items in the questionnaire to perform factor analysis (34). The minimum sample size would thus be 90 for this research compared to our observed sample size of 185. We conducted Bartlett’s sphericity test to examine whether the EFCR item variables correlate and the Kaiser-Meyer-Olkin (KMO) test to assess the strength of the partial correlation between the variables. Both tests indicated that the sample was suitable for factor analysis, with an overall KMO sample adequacy of 0.81 and a *p*-value of < .001 from Bartlett’s sphericity test.

A scree test suggested a two-factor solution to best fit the data. Exploratory factor analysis (EFA) was conducted with an oblique rotation to enable correlation between the latent factors (Supplementary Table S3). Next, items with loadings above 0.4 were retained in the appropriate factors. A visual-cue-related item (“My child gets excited when he/she sees restaurant logos”) was dropped from the second factor, i.e., the auditory food cue responsiveness domain. The goodness-of-fit of the factor structure was evaluated via the Chi-square test for adequate fit, root mean square error of approximation (RMSEA), comparative fit index (CFI), Tucker-Lewis index (TLI), and standardized root mean square residual (SRMR) (35). Based on prior research (35), we considered a non-statistically significant result from the Chi-square test, RMSEA < 0.06, SRMR < 0.08, CFI > 0.95, and TLI > 0.95, as indicators of a relatively good model fit. Since the best solution resulted in two factors, all final analyses included three EFCR scores: one composite score as the mean of all items retained (EFCR-6) and two average scores as the mean across the items within each subscale, separately. Internal consistency was assessed with Cronbach’s and McDonald’s. A threshold of 0.7 was considered acceptable internal consistency. Spearman’s correlations were calculated between the EFCR scores and the set of convergent validity variables and correlates of EFCR. One-year test-retest reliability was examined with average score intraclass correlation (ICC) with a two-way consistency model.

As a sensitivity analysis, confirmatory factor analysis (CFA) was constructed to examine the validity of the original one-factor solution proposed by Masterson and colleagues (13). Second, item loadings were computed for males and females to assess the stability of the factor structure by sex. Further, linear regressions were conducted between correlates of external food responsiveness and each EFCR-6 subscale, adjusting for the other subscale. Lastly, models on PRSs were further adjusted for European ancestry to control for potential confounding by ancestry. All statistical analysis was performed with R version 4.3.2 (36). A *p*-value < .05 was considered as the threshold for statistical significance.

## RESULTS

### Sample Characteristics

Mean age was 10.9 (SD = 1.2) years (N = 185) (Table 1), 42% of the children were female, 90% were white, non-Hispanic, 16% had obesity, and 84% were with European ancestry. Children spent 2.6 (SD = 1.7) hours per day on media. Most parents (84%) had bachelor’s degrees or higher. Thirty-four percent of the families had an annual household income above 145,000 dollars. Children scored 1.87 (SD = 0.48) on the original EFCR scale (Supplementary Table S1).

### Construct Validity

**Figure 1.**
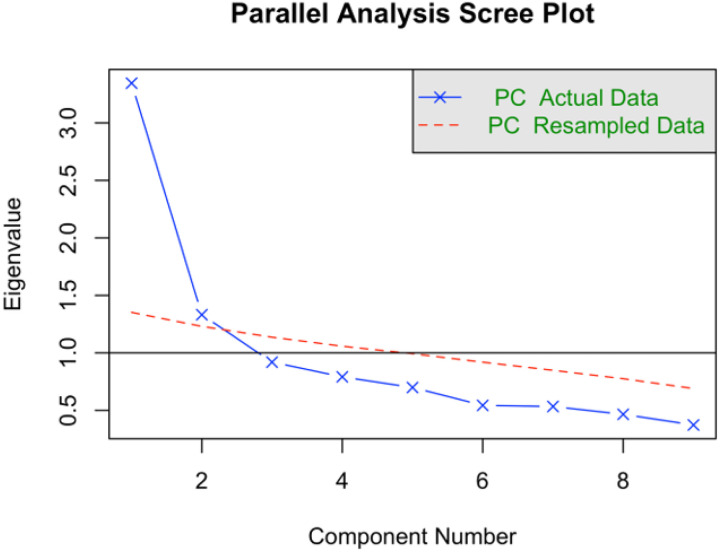
Scree plot from the parallel analysis. *Note*: The scree plot compares eigenvalues from a principal component analysis with the observed data to a principal component analysis from re-sampled data. PC, principal component. Components with observed data eigenvalues above one and exceeding those from re-sampled random data were retained.

The scree plot revealed a two-factor solution, with eigenvalues of 3.34 and 1.32, respectively (Figure 1). This solution retained six items (EFCR-6) with loadings ranging from 0.43 to 0.66 (Table 2). The items were grouped into two subscales: (1) visual food cue responsiveness (EFCR-visual, three items, = 0.74, = 0.74) and (2) auditory food cue responsiveness (EFCR-auditory, three items, = 0.69, = 0.70) (Table 2). The correlation between the two factors was 0.47. This factor structure demonstrated strong fit to the data (CFI = 0.98, TLI = 0.96, RMSEA = 0.06, SRMR = 0.05, = 13.31, degree of freedom = 8, *p*-value from the Chi-square test = 0.10, Table 2). The reduced scale, EFCR-6, showed acceptable internal validity (= 0.73, = 0.73). The average score was 1.90 (SD = 0.54) in this preadolescent sample (Table 1). The scores were similar regarding child sex, race/ethnicity, and household socio-academic status (Supplementary Table S4). Sensitivity analysis showed that item loadings of this shortened version were similar in females and males (Supplementary Table S5). Scores on EFCR-6 correlated strongly with the original EFCR scale (*r* = 0.94, *p* < 0.0001).

### Test-Retest Reliability

Participants in the test-retest sample (N = 96) had demographics similar to those in the baseline sample (Supplementary Table S6). Participants scored 1.88 (SD = 0.65) on the reduced scale at one-year follow-up (Supplementary Table S7). The test-retest reliability was 0.74, assessed via ICC (95% CI: 0.61, 0.83) (Supplementary Table S7).

### Convergent Validity

The reduced scale, EFCR-6, converged with food approach appetitive traits (Table 3). EFCR-6 was positively associated with CEBQ food responsiveness (*r* = 0.41, *p* < 0.01) and emotional overeating (*r* = 0.40, *p* < 0.01). Correlations with the CEBQ food approach appetitive traits were stronger when considering the auditory cue responsiveness factor scores. No associations were found for EAH or BMI.

### Associations with Correlates of External Food Cue Responsivity

EFCR-6 correlated with household factors and general parenting (Table 4). More household chaos correlated with higher EFCR-6 scores (*r* = 0.28, *p* < 0.01). Instrumental feeding (*r* = 0.15, *p* = 0.04) and restrictive feeding (*r* = 0.42, *p* < 0.01) were positively associated with EFCR-6. Responsive parenting (*r* = −0.16, *p* = 0.03) and demanding parenting (*r* = −0.15, *p* = 0.04) were inversely associated with EFCR-6. Notably, children with neglectful parents (mean = 2.05, SD = 0.55) scored higher on EFCR-6 than those with authoritative parents (mean = 1.78, SD = 0.53) (*p* = 0.03 via between-group comparison using Wilcoxon rank sum test) (Supplementary Table S4). Child media use positively correlated with EFCR-6 (*r* = 0.17, *p* = 0.02). No association was found for child obesity genetic risk scores or trait impulsiveness (Table 4).

Associations with child trait impulsivity, media use, and parenting practices were stronger when considering the visual cue responsiveness factor (Table 4). However, the auditory cue responsiveness factor had stronger correlations with household chaos (*r* = 0.25, *p* < 0.01) and 265 SNP pediatric-specific PRS (*r* = 0.18, *p* = 0.02). Further adjusting for European ancestry rendered similar results (Supplementary Table S8). The associations in Table 4 remained similar after adjusting for the two subscales simultaneously (Supplementary Table S9).

## DISCUSSION

The current study validated the factor structure of the External Food Cue Responsiveness scale in 185 high-income US children aged 9 to 12. The study provided initial evidence on the validity and one-year test-retest reliability of the six-item version of the EFCR scale for children in middle childhood. Further research should replicate this factor structure in samples with more diverse sociodemographic profiles.

The factor analysis did not replicate the one-dimensional structure proposed in the original study in 2- to 5-year-old children (Supplementary Table S1) (13). A two-dimensional six-item structure demonstrated a satisfactory fit to this preadolescent sample (CFI = 0.98, TLI = 0.96, RMSEA = 0.06, SRMR = 0.05, = 13.31, degree of freedom = 8, *p*-value from the Chi-square test = 0.10, Table 2). Factors align with visual and auditory cue responsiveness. Two items (“Q4: My child asks for foods or drinks that other kids eat” and “Q9: My child expects a snack when in the car”) had loadings below 0.4, potentially because these behaviors are less applicable to older children (Supplementary Table S3). Another item about visually cued eating, “Q8: My child gets excited when he/she sees restaurant logos,” was removed from the second factor, which reflects the child’s reaction to sound-related food cues. Item loadings were similar in females and males, indicating factor structure stability by sex (Supplementary Table S5). These findings support the validity of a six-item version of the EFCR scale in preadolescents. Additional research should cross-validate this factor structure in more diverse preadolescent cohorts.

The visual food cue responsiveness subscale examines eating responses cued by food packaging and placement. Experimental studies found that visual cues, such as packaging colors (37), can affect children’s food preferences. For instance, children may associate certain cartoon characters with positive emotions, and seeing these characters on food packaging may trigger emotional responses and influence their preferences (18). Another systematic review found that larger package sizes and illustrations depicting exaggerated portions were associated with more food consumption in young children (17). However, most existing research on visual cues and child eating responses occurred in a laboratory, and research on visually cued eating responses is limited in older children (17). Future research should explore how child responsivity to visual food cues translates into overeating and relates to weight status in this age group.

The auditory food cue responsiveness (EFCR-auditory) subscale in the EFCR-6 has three items on sound and social cues, such as hearing snacks being opened and people talking about food. We found that household chaos was positively related to higher children’s reactivity to auditory food cues (Table 4). This finding aligns with our hypothesis because household chaos is characterized by excess noise, a large number of people living together, and a lack of routines (38), which can increase the opportunity for children to be exposed to sound-related stimuli, such as cooking sounds or people discussing foods. Additionally, the highly unpredictable environment may make children more responsive to eating cues (39). However, our correlation analysis was exploratory and subject to potential unadjusted confounding. Longitudinal studies should assess how household chaos affects EFCR. We also identified a positive association between the EFCR-auditory and the 265 SNP pediatric-specific PRS, which aligned with Renier et al.’s study that linked genetic obesity risk to higher CEBQ-FR (9). Further research should examine whether the genetic influence on food cue responsiveness would be specific to sound-related food cues.

The second factor, visual food cue responsiveness, EFCR-visual, showed stronger associations with modifiable environmental and behavioral factors. Consistent with our hypothesis, this factor was positively related to child media use. Children who spend more time on screen-related activities potentially have more exposure to targeted food marketing and thus develop stronger reactions to these visual cues (40). In addition, we observed that less responsive parenting and more coercive feeding were related to higher EFCR-visual (Table 4). These associations remained consistent after further adjusting for the correlations of EFCR-auditory with responsive parenting and coercive feeding (Supplementary Table S9). These findings suggest that child reactivity to visual food cues is potentially learned and modifiable. If so, promoting more supportive and non-coercive parenting practices may help cultivate healthy appetitive traits in children. Further longitudinal and intervention studies should explore whether improving the quality of parent-child interaction and home environment would reduce a child’s propensity to react to food cues.

We did not observe a correlation between the adapted EFCR scale and lab-observed EAH in our sample. This lack of correspondence between the two metrics reveals potential measurement errors in the parent reports on their child’s reactions to food cues. Although parent input is commonly used as a proxy in research assessing appetitive traits in children and adolescents (6), it is unclear how reliable parent perception is in reflecting the child’s experience. Further research should develop a self-report version of the EFCR scale. Examining the agreement between the two versions will offer insight into the suitable age to administer the EFCR scale to children.

Lastly, no associations were found between the adapted EFCR scale and child weight status, which differed from the study in young children (13). However, weight status was parent-reported in that study (13). Potentially, the observation period was not long enough to observe a cumulative change in child adiposity. It is also possible that external food cue reactivity affects child adiposity indirectly through increased food approach appetitive traits, such as emotional overeating. Longitudinal studies and mediation analysis should interrogate how external food cue reactivity translates into unhealthy eating patterns and increased obesity risk in children.

### Strengths and Limitations

In this study, we examined the factor structure of the External Food Cue Responsiveness scale and proposed a shortened version, EFCR-6, for children in middle childhood. The EFCR-6 scale demonstrated satisfactory model fit, construct validity, internal consistency, and reliability in this high-income preadolescent cohort. However, this study has limitations, including limited representativeness of the study sample, the lack of a second independent sample to cross-validate the factor structure, and potential measurement error and information bias in parent- and child reports. Children in our study sample were primarily white, non-Hispanic, and of higher socioeconomic status, which may limit the generalizability of our findings. Also, common method bias may exist since the same parent provided primary outcomes and covariates data. This issue may bias the results in the correlation analysis. Future studies should aim to obtain measures from multiple sources or at different time points to reduce this issue. Lastly, the observed associations in the convergent and construct validity analysis were exploratory and subject to unadjusted confounding and need further investigation.

## CONCLUSIONS

This study offered initial evidence on the validity and reliability of the six-item version of the External Food Cue Responsiveness Scale (EFCR-6) in preadolescents. Future research should validate the scale in more diverse cohorts and develop a child-report version to assess external cue reactivity in preadolescents, an understudied population. As food cues become common in the current food environment, a brief questionnaire on children’s responsiveness to these stimuli will facilitate research and clinical practices on children’s externally cued eating.

## Supplementary Material

Supplementary Files

This is a list of supplementary files associated with this preprint. Click to download.

- EFCRBMCSupplementalMaterial.pdf

## Figures and Tables

**Table 1.** Sample characteristics (N = 185).

	Mean	SD
**Child age (years)**	10.90	1.19
**Child BMI-z**	0.48	1.00
**EFCR-6 score**	1.90	0.54
	n	%
**Child sex (N = 184)**		
Male	107	58
Female	77	42
**Child race/ethnicity**		
White, non-Hispanic	167	90
Others	18	10
**Child weight status**		
With overweight	27	14
With obesity	30	16
**Child media use (hours per day)**		
<1	31	17
1 and <2	44	24
and <3	46	25
and <4	40	22
4 and above	24	13
**Household annual income ($)**		
<65,000	24	13
65,000–145,000	99	54
145,000–225,000	44	24
Over 225,000	18	9
**Parental education**		
Without a bachelor’s degree	30	16
Bachelor’s degree	51	28
Professional or graduate school	104	56

*Note*: SD, standard deviation. EFCR-6, the External Food Cue Responsiveness scale, short version (range: 1 – 4, with 4 being the most reactive to external food cues). BMI-z, age- and sex-adjusted body mass index z-score based on the 2000 CDC growth charts. Anthropometry was missing for one participant and was imputed with the sample mean. Media use was missing for one participant and was imputed with the sample mean. Household income had eight missing values, and parental education had one missing value, both imputed with the sample modes.

**Table 2.** Psychometric properties of the six-item External Food Cue Responsiveness scale (EFCR-6) in preadolescents (N=185).

**Fit measure**	
The Chi-squared test statistic (degree of freedom)	13.31 (8)
*P*-value from the Chi-squared test	0.10
Comparative fit Index (CFI)	0.98
Tucker-Lewis index (TLI)	0.96
Root mean square error of approximation (RMSEA)	0.06
Standardized root mean square residual (SRMR)	0.05
**Cronbach’s**	
Overall	0.73
Factor 1	0.74
Factor 2	0.69
**McDonald’s**	
Overall	0.73
Factor 1	0.74
Factor 2	0.70
**Factor correlation**	0.47
*Factor 1: visual food cue responsiveness*	**Standardized loadings**
My child wants snacks at check-out aisles	0.66
My child likes certain snacks because of the packaging.	0.56
My child points out snack or drink vending machines	0.58
*Factor 2: auditory food cue responsiveness*	
My child gets excited by the sound of food cooking.	0.49
My child wants to eat if he/she hears a snack being opened.	0.62
My child wants to eat when people talk about food.	0.43
**One-year test-retest reliability (ICC, N = 96)**	0.74

*Note*: All items are prefaced with “My child…”. Item responses are anchored on a four-point Likert-scale: rarely (1), sometimes (2), often (3) or a lot (4). The final score is the average score across six items, ranging from 1 to 4, with 4 being the most reactive to external food cues. ICC, average score intraclass correlation computed with a two-way consistency model.

**Table 3.** Convergent validity of the six-item External Food Cue Responsiveness scale (EFCR-6) in preadolescents (N = 185).

			EFCR-6		Visual Food Cue Responsiveness Subscale		Auditory Food Cue Responsiveness Subscale	
Convergent Validity Variable	Mean	SD	*r*	*p*	*r*	*p*	*r*	*p*
CEBQ food responsiveness	2.58	0.74	0.41	<0.01	0.23	<0.01	0.44	<0.01
CEBQ emotional overeating	2.21	0.66	0.40	<0.01	0.28	<0.02	0.38	<0.01
CEBQ enjoyment of food	3.94	0.59	0.24	<0.01	−0.02	0.82	0.45	<0.01
EAH (calories)	14.70	173.76	−0.01	0.85	−0.01	0.84	−0.03	0.71
BMI z-score	0.48	1.00	0.07	0.37	0.04	0.62	0.06	0.40

*Note*: EFCR-6, the six-item External Food Cue Responsiveness scale. CEBQ, Child Eating Behavior Questionnaire (range: 1 to 5). EAH, the difference in the caloric intake during the eating in the absence of hunger experiment between food advertisement and toy advertisement condition. BMI z-score, age- and sex-adjusted body mass index based on the CDC 2000 growth charts. This table presents Spearman’s correlation coefficients of the EFCR-6 scale or subscale with convergent validity variables.

**Table 4.** Associations between the six-item External Food Cue Responsiveness scale (EFCR-6) and correlates in preadolescents (N=185).

			EFCR-6		Visual Food Cue Responsiveness Subscale		Auditory Food Cue Responsiveness Subscale	
Correlates of EFCR	Mean	SD	*r*	*p*	*r*	*p*	*r*	*p*
Child trait impulsiveness	0.37	0.20	0.11	0.13	0.25	<0.01	−0.08	0.30
Child media use, hours/day	2.59	1.72	0.17	0.02	0.27	<0.01	−0.02	0.74
Instrumental feeding	2.14	1.11	0.15	0.04	0.17	0.02	0.07	0.36
Restrictive feeding	2.96	0.90	0.42	<0.01	0.40	<0.01	0.26	<0.01
Demanding parenting	11.70	2.38	−0.15	0.04	−0.16	0.03	−0.09	0.23
Responsive parenting	16.10	2.78	−0.16	0.03	−0.22	<0.01	−0.04	0.55
Household chaos	28.50	7.08	0.28	<0.01	0.19	0.01	0.25	<0.01
The 97 SNP PRS	NA	NA	0.01	0.93	−0.01	0.88	−0.01	0.94
The ~2 million SNP PRS	NA	NA	0.03	0.65	−0.02	0.81	0.08	0.29
The 265 SNP pediatric-specific PRS	NA	NA	0.09	0.21	−0.02	0.77	0.18	0.02

*Note*: SD, standard deviation. SNP, single nucleotide polymorphisms. PRS, polygenic risk score (N = 178). EFCR-6, the six-item External Food Cue Responsiveness scale. This table presents Spearman’s correlation coefficients of the EFCR-6 scale or subscale with each correlate of EFCR. Trait impulsiveness was assessed via Eysenck’s Impulsivity Inventory (N = 184, range: 0 to 1; higher scores indicate more dispositional impulsiveness). Child media use was collected via parental reports. Feeding practices were assessed via the Comprehensive Feeding Practices Questionnaire (CFPQ, range: 1 to 5; higher scores indicate more instrumental or restrictive feeding behavior). Parenting style was assessed via the Authoritative Parenting Index (API, range: 4 to 16 for demandingness; 5 to 20 for responsiveness; high scores indicate more demanding or responding parenting). Household chaos was evaluated via the Confusion, Hubbub, and Order Scale (CHAOS, N = 184, range:15 to 60, with 60 being the most chaotic household).

## Data Availability

The datasets used and/or analyzed during the current study are available from the corresponding author upon reasonable request.

## Abbreviations

EFCR: the External Food Cue Responsiveness scale
EFA: exploratory factor analysis
ICC: intraclass correlation
CEBQ: Child Eating Behavior Questionnaire
EAH: eating in the absence of hunger
EFCR-6: the short version of the External Food Cue Responsiveness scale
EFCR-auditory: the auditory food cue responsiveness subscale
EFCR-visual: the visual food cue responsiveness subscale
CIRO: the Cue-Influencer- Reactivity-Outcome model
FR: food responsiveness
PRS: polygenic risk score
CFA: confirmatory factor analysis
DEBQ-c: Children’s Dutch Eating Behavior Questionnaire
EE: external eating
MRI: magnetic resonance imaging
EOE: emotional overeating
EF: enjoyment of eating
BMI: body mass index
CFPQ: Comprehensive Feeding Practices Questionnaire
API: Authoritative Parenting Index
CHAOS: Confusion, Hubbub, and Order Scale
SNP: single nucleotide polymorphism
KMO: the Kaiser-Meyer-Olkin test for sampling adequacy
RMSEA: root mean square error of approximation
CFI: comparative fit index
TLI: Tucker-Lewis index
SRMR: standardized root mean square residual
SD: standard deviation
PC: principal component
